# MAPK/JNK signalling: a potential autophagy regulation pathway

**DOI:** 10.1042/BSR20140141

**Published:** 2015-06-11

**Authors:** Yuan-Yuan Zhou, Ying Li, Wei-Qin Jiang, Lin-Fu Zhou

**Affiliations:** * Medical Biotechnology Laboratory, Zhejiang University, Hangzhou 310058, China; † Children's Hospital Affiliated to the Medical College of Zhejiang University, 268 Kaixuan Road, Jianggan District, Hangzhou 310029, China; ‡ Cancer Center, First Affiliated Hospital, Zhejiang University School of Medicine, 79 Qingchun Road, Shangcheng District, Hangzhou 310003, China

**Keywords:** autophagic cell death, autophagy, B-cell lymphoma 2 (Bcl-2), Beclin1, mitogen-activated protein kinase (MAPK)/c-Jun NH2-terminal kinase (JNK)

## Abstract

Autophagy refers to a lysosomal degradative pathway or a process of self-cannibalization. This pathway maintains nutrients levels for vital cellular functions during periods of starvation and it provides cells with survival advantages under various stress situations. However, the mechanisms responsible for the induction and regulation of autophagy are poorly understood. The c-Jun NH2-terminal kinase (JNK) signal transduction pathway functions to induce defence mechanisms that protect organisms against acute oxidative and xenobiotic insults. This pathway has also been repeatedly linked to the molecular events involved in autophagy regulation. The present review will focus on recent advances in understanding of the relationship between mitogen-activated protein kinase (MAPK)/JNK signalling and autophagic cell death.

## Abstract (precis)

Herein, MAPK/JNK signalling is proposed as a potential autophagy regulation pathway for the transcription-dependent or independent role.

## INTRODUCTION

In 1963, the term autophagy was first coined by Christian de Duve, not long after the discovery of lysosome [1]. The term is derived from Greek, where ‘auto’ means oneself and ‘phagy’ means to eat. It refers to the cellular degradation process in which cytoplasmic organelles are transported into lysosomes [2,3]. Depending upon physiological functions and ‘cargo’ transportation mechanisms, autophagy can be divided into at least three types: chaperone-mediated autophagy (CMA), microautophagy and macroautophagy. It is widely believed that macroautophagy is the primary type and draws research interest. Thus, the generic term autophagy in most cases refers to macroautophagy.

Autophagy is an evolutionarily conserved process found in eukaryotes ranging from yeast to mammals [4]; it is the only mechanism for degrading large structure, including macromolecules such as protein polymers and organelles and it is self-cannibalization mechanism. Under homoeostatic conditions, autophagy mainly acts as a ‘housekeeper’, providing ‘waste management and treatment’ services that remove injured cellular components which might become toxic. Such cellular maintenance is important, especially in resting cells and terminally differentiated cells where injured components cannot be diluted through cell proliferation [3,5]. When cells experience starvation, autophagy can provide them with nutrition that maintains their viability. Autophagy can be induced by a number of stressors, acting to degrade protein polymers, oxidized lipids, injured organelles, as well as intracellular pathogens [6].

Autophagy is an effective internal regulatory mechanism that allows biological organism to adapt to different environments. It can protect organisms from metabolic stress, act as a ‘housekeeper’ and it, in part, considered to be functioned in decisions of cell survival and death [2]. Activation of autophagy has potential risks: it has the potential to rescue diseased cells from death, such as tumour cells [7,8]. Some studies in the past years indicated that autophagy might be closely related with a series of neurodegenerative diseases, liver diseases, myopathy, heart diseases, tumour progression, aging, infection, immunization and inflammatory diseases [2].

There are more than 30 different autophagy-related (ATG) genes in yeast, as identified by genetic screening, most of which have mammalian homologues. There are 15 ‘core’ ATG genes that encode primary elements for ATG membrane biotransformation and are required in three ATG pathways, including starvation-induced autophagy, the Cvt (cytoplasm-to-vacuole targeting) pathway and pexophagy pathway(an autophagic degradation pathway for peroxisomes in yeast) [9]. Among the ‘core’ ATG genes, *Atg5* and *Atg7* play a significant role in the Atg12-conjugation system and *Lc3* (light chain 3), mammalian orthologous gene of *Atg8* and encodes MAP1LC3 (microtubule-associated protein 1 light chain 3), is vitally involved in the LC3/Atg8-conjugation system in the elongation step of autophagosome formation process [3,10].

The mammalian target of rapamycin (mTOR) and PI3K (phosphoinositide 3-kinase)/Akt, serine/threonine kinase, [also named protein kinase B (PKB)] pathways are considered primary autophagy regulatory pathways and are extensively researched [11,12]. Previous studies indicated that the c-Jun NH2-terminal kinase (JNK) pathway may also play an important role in various forms of autophagy, for instance incidences of nutrition deficiency, cytokines and growth factors decreases and neurotoxic drugs [13–16]. Therefore, a clear understanding of how the mitogen-activated protein kinase (MAPK)/JNK signal pathway influences autophagy can lead to characterization of the underlying molecular mechanisms. Elucidation of the signalling cascades that the regulate autophagy and its mechanisms will be highly beneficial to disease treatment and prevention.

## MAPK/JNK SIGNALLING PATHWAY

### MAPK families

The MAPK signal transduction pathway is one of the most important regulatory mechanisms in eukaryotic cells. Its signal transduction occurs via sequential phosphorylation of MAPKKK (mitogen-activated protein 3 kinase), MAPKK (mitogen-activated protein 2 kinase) and MAPK. MAPK, a highly conservative, serine/threonine protein kinase and is part of a critical signal transduction system. Six MAPK sub-families have been cloned and identified in mammalian cells; they are JNK1/2/3, extracellular signal-regulated kinase (ERK)1/2, p38MAPK (p38 α/β/γ/δ), ERK7/8, ERK3/4 and ERK5/BMK1 (big MAP kinase 1) [17]. After activation by upstream kinases, different sub-families regulate various physiological processes in cells, including inflammation, stress, cell growth, cell development, differentiation and death, through multiple substrates like phosphorylation transcription factors, cytoskeleton-associated cells and enzymes [18].

### JNK pathway and downstream substrates

JNKs were initially identified as the stress-activated protein kinases (SAPKs) in the mouse liver treated with cycloheximide to induce inflammation and apoptosis [19]. It was renamed to emphasize its relationship with c-Jun, a phosphorylation-activated transcription factor. There are three genes that encode JNKs in mammals: *Jnk1*, *Jnk2* and *Jnk3*, among these, *Jnk1* and *Jnk2* are widely expressed *in vivo* whereas *Jnk3* is expressed in the brain, heart and testis.

JNKs are activated by a number of stressors, including UV irradiation and oxidative stress, which can induce apoptosis or growth inhibition [20,21]. MKK4 and MKK7, the upstream kinases (namely, MAP2K) of JNK pathway, are activated by different upstream MAP3K respectively [17]. Once activated, JNKs translocate from cytoplasm to nucleus [22]. The downstream targets of JNK include the transcription factor c-Jun, which translocates to the nucleus after JNK mediated phosphorylation. c-Jun is best known to regulate expression of pro-apoptotic or anti-apoptotic genes *Bax* (Bcl2-associated X protein) and *Bcl-2* (B-cell lymphoma 2) [23,24]. When activated, JNK phosphorylates serine residues 63 and 73 at c-Jun N-terminal thus activating c-Jun and enhancing its transcriptional activity [18]. Other reports have shown the association between JNK and c-Jun acts to stabilize c-Jun in addition to activating it [25].

## ROLE OF BECLIN1 IN REGULATION OF AUTOPHAGY

### Beclin1 and biological response

Beclin1 was initially cloned through yeast two-hybrid screening in 1998 and defined as a novel curve-helix protein that interacts with Bcl-2, which could be encoded by apoptosis-inhibiting gene *Bcl-2* [26]. Subsequent research has revealed autophagy activity in tumours cells correlates with *Beclin1* expression. It is believed that Beclin1, an important ATG protein, its yeast homologue, autophagic gene *Atg6/Vps30* (vacuolar protein sorting), is a ‘core’ element in membrane formation. Beclin1 recruits and activates the hVps34 protein; they form a core complex of Beclin1–hVps34–Vps15. This complex plays a vital role in pro-autophagosomal protein structure formation/phagophore assembly site (PAS) [27,28].

When discussing autophagy and its regulation pathway, we divide the process into a series of stages, without clear boundary: formation of autophagosome, fully functional autophagy systems (maturation and fusion with the lysosomal compartment) and related signal pathways which perform a number of functions [12] (Figure 1). Autophagy begins with the nucleation and isolation of membrane/phagophore, the membrane then extends and closes into an autophagosome. During the process, Beclin1 regulates the formation and maturation of autophagosome through the formation of different complexes. The Atg14L complex (Beclin1–hVps34–Atg14L) can promote nucleation of isolated membranes, whereas the UV irradiation resistance-associated gene protein (UVRAG) complex (Beclin1–hVps34–UVRAG) positively regulate maturation of autophagosome. Conversely, when Beclin1 complexes with Rubicon, the Rubicon–UVRAG complex (Beclin1–hVps34–UVRAG–Rubicon) formed acts to down-regulate autophagy [12,29,30] (Figure 1).

**Figure 1:**
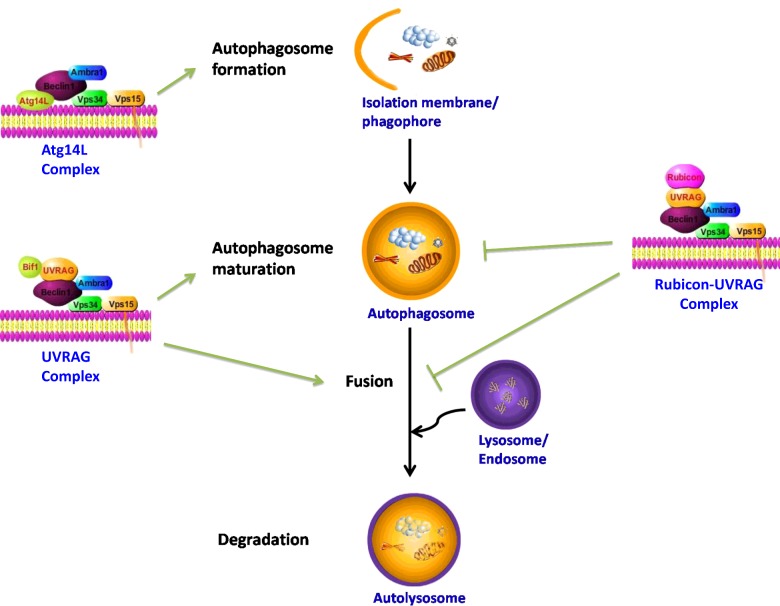
Schematic depiction of the regulation function of three Beclin1 containing complexes in different stages of autophagy The drawing is modified from [12]: Yang, Z. and Klionsky, D.J. (2010) Mammalian autophagy: core molecular machinery and signaling regulation. Curr. Opin. Cell Biol. **22**, 124–131, [30]: Matsunaga, K., Saitoh, T., Tabata, K., Omori, H., Satoh, T., Kurotori, N., Maejima, I., Shirahama-Noda, K., Ichimura, T., Isobe, T., Akira, S., Noda, T. and Yoshimori, T. (2009) Two Beclin 1-binding proteins, Atg14L and Rubicon, reciprocally regulate autophagy at different stages. Nat. Cell Biol. **11**, 385–396 and [40]: Levine, B., Sinha, S. and Kroemer, G. (2008) Bcl-2 family members: dual regulators of apoptosis and autophagy. Autophagy **4**, 600–606. Mammalian autophagy proceeds through a series of stages, including formation of autophagosome, autophagosome maturation via docking and fusion with a lysosome/endosome, breakdown and degradation of the autophagosome inner membrane and cargo by lysosomal proteases and recycling of the resulting macromolecules. There are at least three class III PI3K complexes that are involved in autophagosome formation and maturation. The Atg14L complex (Beclin1–hVps34–Atg14L) functions in autophagosome formation. The UVRAG complex (Beclin1–hVps34–UVRAG) is required for the maturation of autophagosome, whereas the Rubicon–UVRAG complex (Beclin1–hVps34–UVRAG–Rubicon) negatively regulates this process.

### The Beclin1 domains and interacting proteins in autophagy

There are several domains found in Beclin1 that are relevant to its role in autophagy. At the N-terminus amino acids 114–123 make up a BH3 domain (Bcl-2-homology-3 domain), residues 144–269 form a central coiled-coil domain (CCD) and residues 244–337 make an evolutionarily conserved domain (ECD). Bcl-2 interacts with BH3 domains of Beclin1, UVRAG with its CCD and PI3K III with ECD and CCD [31].

Beclin1 acts as a platform protein for recruiting hVps34 (PI3KC3), Vps15 (a congener of p150) and activating molecule in Beclin1-regulated autophagy (Ambra1). It also forms a core complex with PI3K III and it interacts with different activators including UVRAG/Bax-interacting factor-1 (Bif-1), Atg14 (also named Atg14L) and Rubicon (a RUN domain and cysteine-rich domain containing). All of these co-operative binding interactions participate in the regulation process of mammalian cells at different stages of autophagy (Figure 1) [12,30,32–36].

### Bcl-2 inhibits Beclin1-dependent autophagy

Beclin1 is a BH3-only domain protein and the first identified mammalian autophagy protein [37]. It is known to interact with Bcl-2 family anti-apoptosis proteins, primarily Bcl-2 and Bcl-XL, through its BH3 domain and these interactions inhibit Beclin1 activity. Other BH3-only proteins and BH3 simulants can induce autophagy through competitive inhibition of interactions between Beclin1 and Bcl-2/Bcl-XL [38]. Studies have indicated that Bcl-2 as well as viruses can inhibit Beclin1-dependent autophagy in yeast and mammalian cells [39]. Bcl-2 can inhibit formation of the Beclin1–hVps34–PI3K complex and activity of Beclin1-related PI3K III through interactions with Beclin1 and as a result, inhibit autophagy (Figure 2). The transgenic expression of Bcl-2 in mouse myocardial cells reduced starvation-induced autophagy. These results demonstrate Bcl-2 can inhibit autophagy *in vivo* as well as *in vitro* [39].

**Figure 2:**
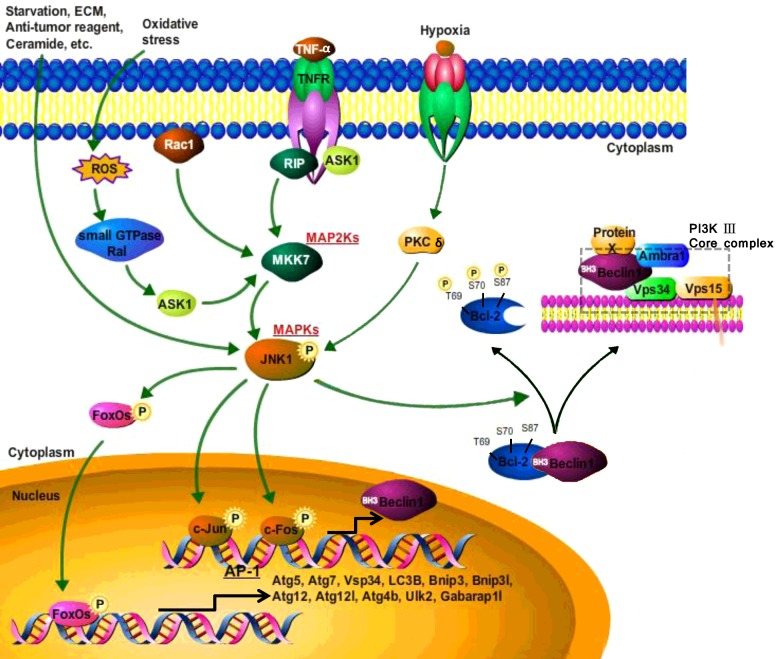
Proposed mechanism of JNK-mediated autophagy MAPK/JNK signalling cascades involved in the regulation of autophagy. Autophagy is induced in response to the absence of amino acids compared with other stresses and regulated by a complicated signalling network delivering to JNK1 (green arrowheads). Constitutively-activated JNK1 leads to Bcl-2 phosphorylation at the amino acid residues (Thr^69^, Ser^70^ and Ser^87^), which dissociates Bcl-2 from Beclin1 and constitutes the Beclin1-related PI3K III complexes (black cambered arrowheads) and as a result, regulates autophagy. The complexes (Beclin1–hVps34–protein X) play stimulatory or inhibitory roles in different stages of autophagy according to protein X (mainly detailed in Figure 1). When activated, JNK phosphorylates and thus activates c-Jun/c-Fos, enhancing its transcriptional activity of Beclin 1.Activated JNK also induces FoxOs nuclear localization and increases its activity to regulate transcription of other ‘core’ ATG genes.

In order to understand the internal relationship between autophagy and cell survival, researchers have proposed a theoretical model: Beclin1–Bcl-2 complex can act as a rheostat in autophagic regulation. Autophagy is a necessary adaptive response to nutrition deficiencies and other types of cell stress. Without autophagy cells are less resilient and loose viability when exposed to deleterious stimulation. Pattingre et al. [39] concluded the Beclin1–Bcl-2 complex might act as rheostat, maintaining the proteins responsible for activating autophagy in a state of ready, ensuring a rapid response when cellular stressors are encountered.

Subsequent research indicates the regulation of Bcl-2/Bcl-XL and Beclin1 interactions governs the autophagy response to different cell stresses [40]. Furthermore, post-translational modifications of Bcl-2 or Beclin1 have been shown to alter the interaction between Bcl-2/Bcl-XL–Beclin1. Other proteins have been shown to affect Beclin1 activity. BH3-only proteins can interfere with Bcl-2/Bcl-XL–Beclin1 interactions adding another layer Beclin1 regulation. In addition, membrane-anchored receptors or their ligand proteins might be able to regulate the interaction between Bcl-2/Bcl-XL–Beclin1. The regulation mechanisms which co-ordinate autophagic activity appear to act interactions between Bcl-2/Bcl-XL and Beclin1, at least to a certain degree [28]. Disruption of the Bcl-2/Bcl-XL and Beclin1 interaction is perhaps a mechanism that promotes autophagy and cell survival when the organism experiences cellular stresses, such as nutritional deficiency, hypoxia or separation from the extracellular matrix (Figure 2) [7,41].

## ROLE OF JNK IN AUTOPHAGY REGULATION

### JNK and autophagic cell death

JNK participates in multiple stimulation-induced autophagic events, including endoplasmic reticulum stress [42], caspase (cysteine aspartate-special proteases) inhibition [43], insulin-like growth factor-1 treatment and exposure to tumour necrosis factor-α (TNF-α) [16]. Moreover, JNK has been related to autophagic-induced cell death. The concept of autophagic cell death was redefined as a modality of non-apoptotic or necrotic programmed cell death (PCD) in which autophagy serves as a cell death mechanism [44]. When cells act to down-regulate oncogenic gene expression, oncogenic stress can trigger autophagic cell death as a defence mechanism [45].

Agonistic anti-TR2 (tumor necrosis factor (TNF)-related apoptosis-inducing ligand (TRAIL) receptor 2) single-chain fragment variable (scFv), HW1-induced autophagic cell death mainly functions through JNK pathway [46]. Puissant et al. [47] reported that phytoalexin resveratrol (RSV) could induce autophagy in chronic myeloid leukaemia (CML) through JNK-dependent p62 accumulation, as well as through over-expression of JNK-mediated p62 and AMPK (AMP kinase) activation. Research on acute toxic neuronal death in the hippocampus of mice done by Borsello et al. [15] discovered neuronal death was neither apoptosis nor necrosis but was in fact characteristic of autophagic neuronal death. In addition, JNK pathway activation, c-Jun selective phosphorylation and c-Fos selective up-regulation were observed in the present study. All these phenomena were inhibited by JNK and the cell-permeable inhibitors of its downstream substrates. These results indicate the neuronal cell death observed here was autophagic in nature and thus was regulated by JNK pathway [15].

It has been reported anti-tumour reagents, including arsenic trioxide and Δ9-THC (tetrahydrocannabinol) can induce autophagy without triggering caspase-dependent apoptosis [48–50]. In the past, it has been proposed that caspase-independent autophagic cell death is related to ROS and/or the JNK signalling pathway [15,51]. Autophagic cell death induced by dopaminergic-specific neurotoxin MPP+ (1-methyl-4-phenyl-pyridinium ion) treatment or oxidative stress is activated by pro-death signals mediated by ROS-dependent JNK. This autophagic response is inactivated by pro-survival signal of AKT/mTOR pathway regulation. These results indicated MPP+-induced cell death is an autophagic instead of apoptotic response and such response is aroused by oxidative stress and mediated by JNK (activation) and AKT/mTOR (inactivation) signal pathway [52]. Other research indicates that, ROS-induced JNK and ERK activation induces both autophagy and apoptosis, that is to say, ERK and JNK can trigger drug-induced autophagy and apoptosis at the same time. ROS has also been shown to be closely related with this signal pathway [53]. Xie et al. [54] found autophagy-induced in colon cancer treatment using bufalin, an anti-cancer drug. This process relies on ROS and the JNK pathway, the induction of ROS by bufalin is an upstream event to JNK activation [54] (Figure 2).

### JNK signalling regulates the expression of multiple ATG genes

JNK can modulate autophagy at multiple regulatory levels. Apart from protein phosphorylation, lipid and processing [55–58], autophagy is also probably regulated by other mechanisms, such as ATG gene expression. Wu et al. [59] proposed the importance of JNK gene regulation in autophagy in *Drosophila*. Here, when the small intestine of *Drosophila* was exposed to oxidative stress or artificially activation of the JNK pathway, digestive tract epithelial cells experienced a significant increase in autophagosomes. JNK was observed genetically interacted with ATG genes using EM and histology. Given these researchers concluded oxidative stress could induce autophagy in *Drosophila* digestive tract via the JNK signal pathway and activation of the ATG genes, at least to some degree. Besides, enhancing *Atg1*, *Atg6* or *Atg8a* expression artificially could induce autophagy of cells [55,60,61]. As a result, it is reasonable to believe that the increase in JNK-induced ATG genes expression level might initiate and/or maintain autophagy of stress organs. Researches have shown that JNK regulates autophagy through both cytoplasmic and nuclear events [29]. To date, little is known about how JNK pathway activation, autophagy induction and the expression of ATG genes come together in this context.

Yu et al. [43] defined a new signalling pathway through a morphological study of autophagy. This pathway included receptor-interacting protein (RIP, a serine/threonine kinase), MKK7, JNK and c-Jun. It also required participation of Atg7 and Beclin1, activated autophagy and induced cell death by inhibiting caspase-8. Since the process involved composition of c-Jun and other proteins, it might require transcription of target genes [43] (Figure 2). Wong et al. [53] suggested expression of Atg7, an important mediator for autophagosome formation, could be effectively blocked via inhibition of JNK expression, JNK activation play a role in regulating *Atg7* expression.

Jia et al. [16] reported that Akt and JNK pathways participate in the expression of autophagic gene *MAP1LC3* in response to TNF-α, in vascular smooth muscle cells of atherosclerosis patients. Sun et al. [62] found that human nasopharyngeal carcinoma cells treated with ceramide demonstrated autophagy characteristics and activation of JNK pathway. Inhibition of the JNK pathway blocked ceramide-induced autophagy and up-regulation of LC3 expression [62]. These data provide a new regulatory mechanism of LC3 expression in anti-tumour reagent-induced autophagy. Xie et al. [54] found that JNK pathway participated in bufalin-induced autophagy. In *jnk2*–siRNA knockdown experiments, bufalin-mediated LC3 II levels did not change, but a significant decrease in cell autophagy and cell death was observed. The increases in Atg5 and Beclin1 mRNA and protein levels were also inhibited. These data indicates in colon cancer cells, after bufalin treatment, JNK activation is necessary for up-regulation of *Atg5* and *Beclin1* as well as autophagy-mediated cell death [54].

Further research indicated up-regulation of *Atg5* expression in the wild-type fibroblasts was indispensable for formation of H-ras-induced autophagic vesicle and caspase-independent cell death. Specifically, JNK activation was proved crucial in this process [45]. There were exclusive to JNK activation that ERK and p38 MAPK activation did not result in *Atg5* up-regulation. Therefore, JNK activation can, in response to oncogenic H-ras, up-regulate *Atg5*, autophagy and non-apoptotic cell death signals. In addition, inhibition of c-Jun dramatically inhibited H-ras-mediated induction of *Atg5*, autophagy and cell death [45]. Finally, activation of Rac1/MKK7/JNK/c-Jun signal pathway was crucial for the up-regulation of *Atg5*.

Researchers have reported anti-tumour reagents activated JNK-mediated Beclin1 expression, inducing autophagic cell death in tumour cells [63]. Treatment of human tumour cell lines with sphingolipid ceramide can up-regulate *Becn1* gene expression [64]. Moreover, c-Jun is known to regulate transcription of *Beclin1* [63]. It was also reported that JNK activation induced up-regulation of Beclin1 expression via human death receptor 5-induced autophagic death in human colon cancer cells [65]. Whereas activating the Beclin1-dependent JNK pathway in human hepatoma carcinoma cells, cytoplasmic deacetylase HDAC6 (histone deacetylase 6) was thought to mediate caspase-independent autophagic cell death [66]. These findings further support our conclusion mentioned above. The experiment indicated HDAC6-activated and -mediated autophagic cell death through the Beclin1 and LC3 II pathways. Here, overexpression of HDAC6-activated JNK and enhanced c-Jun phosphorylation. Conversely, induction of Beclin1 expression was inhibited by SP600125 and HDAC6–siRNA knockdown [66].

### JNK regulates autophagy through FoxO-dependent transcription of ATG genes

JNK contains a large number of nuclear and cytoplasmic target genes, most of which are transcription factors, including AP-1 (activator protein 1) family members *Jun*, *Fos* and FOX (forkhead box) O transcription factor *FoxO* [67,68]. FoxOs is characteristic of its winged helix domain and combines with DNA through this domain [69]. FoxO transcription factor in mammals are composed of four members: FoxO1, FoxO3, FoxO4 and FoxO6, but there is only one FoxO isoform in *Drosophila*: dFoxO (dFoxO, *drosophila* FoxO) [70].

Autophagy in *Drosophila*, is a core mechanism similar to those found in yeast and mammals, includes ATG proteins and signalling cascades [71]. Therefore, it is regarded as an effective model for autophagy research. Studies conducted in *Drosophila* have indicated, after oxidative stress treatments, the expression levels of Atg1 and Atg18 raise instantaneously and becomes consistent with JNK peak activation prior to autophagic events. This implies that ATG genes are target genes of the JNK pathway [59]. The dFoxO protein is a member of FoxO transcription factor family, which is regulated downstream of JNK activation [18,72]. For example, it is believed ROS-activated dFoxO is mediated by phosphorylated FoxO [73]. Observations of genetic experiments in *Drosophila* have demonstrated the close relationship between JNK and dFoxO [74,75]. Furthermore, it has been reported that dFoxO is indispensable for autophagy induction [76]. Given these data, it is possible that the influence of JNK on autophagy is mediated via FoxO-dependent ATG genes transcription.

FoxO transcription factor can regulate autophagy. Oxidative stress phosphorylates FoxO4 via small GTPase Ral-activating JNK, thus inducing FoxO4 nuclear localization and increases its activity. However phosphorylation site that JNK acts at is non-conservative in FoxO1, FoxO3 and FoxO6 [77–79]. Lee et al. [80] reported a possible mechanism where FoxO transcription factor directly controlled initiation and maintenance of autophagy. Zhao et al. [81] observed FoxO-mediated induction was not affected by FoxO1 transcriptional activity; as a result, it was proposed cytoplasmic FoxO transcription factor is necessary for inducing autophagy. Down-regulation of FoxO1 inhibits p62 degradation and LC3 II accumulation, resulting in autophagosome decrease [81]. Surprisingly, FoxO1 mutant in cytoplasm of enhanced expression is sufficient to induce autophagy and autophagic cell death [81]. It has also been reported that FoxO1 and Atg7 interaction is indispensable for autophagy induction. The same study indicated only the FoxO1 isoform could form this interaction, however little is known as to how this interaction induces autophagy.

FoxO, in mammals, induces ATG gene expression (Figure 2) [82–84]. FoxO3 is indispensable in starvation-induced autophagy, because it regulates transcription of ATG genes including *Lc3* and *Bnip3* (belong to Bcl2/adenovirus E1B 19 kDa-interacting protein 3). Evidence suggests Bnip3 can regulate FoxO3-induced autophagy [82]. Constitutively-active FoxO3 (ca-FoxO3) has been shown to induce the formation of autophagosome (autophagic vacuoles) in isolated adult mouse muscle fibres and to stimulate expression (exactly described as enhance transcription in mRNA levels) of multiple ATG genes in myotubes, including *Lc3b*, *Bnip3*, *Bnip3l*, *Atg12l*, *Atg4b*, *Ulk2* (unc-51 like autophagy activating kinase 2), *Gabarapl1* (gamma-aminobutyric acid (GABA) A receptor-associated protein-like 1), *Vsp34* and *Beclin1* [83]. Similar gene expression changes occurred in mouse muscle after denervation or food deprivation [82]. Based on these findings, researchers propose the FoxO transcription factor is a major regulator of ATG genes. However, as mRNA levels of these transcription factors increases, no changes in protein levels are detected. Perhaps this occurs due to degradation during the autophagy process, which could be deduced from the detected enhancement of either LC3 conversion and GABARAPL1 protein levels or their lysosomal degradation after infected with ca-FoxO3 viruses. FoxO3 direct binds to the LC3B and GABARAPL1 promoters and increases their production, which are then consumed by lysosomal proteolysis [83]. Therefore, a potential function of FoxO is not to enhance autophagy, but to maintain high levels of autophagy by regulating essential proteins, which are degraded once autophagy is enhanced.

Researchers have demonstrated the functionally diversity of *Foxo* genes in mammals and found an interesting phenomenon: *FoxO1* tends to induce *Atg12* expression and *FoxO3* priors to activating *Lc3* genes. These data indicate isoform-specific regulation of FoxO in autophagy [85–87]. Furthermore, these results emphasize FoxO3, but not FoxO1, is activated when autophagy occurs in ischaemia/reperfusion injuries [88]. Neurons with compound JNK defects (JNK-TKO (triple *Jnk* knockout), which means eliminate *Jnk1*, *Jnk2* and *Jnk3* at the same time) rely on autophagy to survive and this autophagic response is brought about by FoxO-induced *Bnip3* expression. Here Bnip3 can replace the autophagic effector molecule Beclin1 in the inactive Bcl-XL complex. In JNK-TKO neurons, up-regulation of FoxO target genes *Bnip3*, *Lc3b* and *Atg12* was observed for the first time, together with an increase FoxO1 mRNA and protein levels [89]. Xu et al. [89] also reported that JNK is an effective negative regulator for FoxO-dependent autophagy in neurons.

### JNK1 is involved in the post-translational modification of Bcl-2 and mediates the dissociation of Beclin 1 from Bcl-2

The anti-apoptotic protein Bcl-2 and other Bcl family members, such as Bcl-XL can inhibit autophagy [39,90,91], Bcl-2/Bcl-XL combines with Beclin1 through anchoring groove in the BH3 domain; this interaction is lost when autophagy-promoting signalling occurs. Under starvation conditions or after ceramide treatment, JNK1, rather than JNK2, is activated and Beclin1 dissociates from Bcl-2 after the phosphorylation of amino acid residues (Thr^69^, Ser^70^ and Ser^87^) thus inducing autophagy (Figure 2) [13,92,93]. Expression of *Bcl-2* phosphorylation triple mutant (T69A, S70A and S87A) or JNK1-inhibiting expression in *jnk1*^*−/−*^ and *jnk2*^*−/−*^ *MEFs* (mouse embryonic fibroblasts) prevent dissociation of Bcl-2 and Beclin1 in starvation conditions thus inhibit autophagy. Conversely, constitutively-activated JNK1 leads to constitutive Bcl-2 phosphorylation, dissociate Bcl-2 from Beclin1 and stimulated autophagy [13,93]. Jung et al. [94] also reported, Bcl-2 phosphorylation was mediated by JNK activation, whether through RelB (avian reticuloendotheliosis viral (v-rel) oncogene related B) activity or via direction JNK action [94]. Accumulation of Bcl-2, in the phosphorylated form, was also observed [94].

Researches have shown after short periods (4 h) of nutrition deprivation a small amount of Bcl-2 phosphorylation causes dissociation of Bcl-2–Beclin1 complex. There was no dissociation of the Bcl-2–Bax complex observed under these conditions. After 16 h of nutrition depravation, Bcl-2 phosphorylation reached its highest level, the Bcl-2–Bax complex dissociated under these conditions, thus activating caspase3 and triggering apoptosis [92]. In order to explain how cells regulate the interaction between autophagy and apoptosis through JNK 1-mediated Bcl-2 phosphorylation, Wei et al. [92] proposed a model according to research described above. When rapid Bcl-2 phosphorylation took place first, it promoted cell survival through dissociation of the Bcl-2–Beclin1 complex and activated autophagy; when autophagy could no longer ensure cell survival, Bcl-2 phosphorylation become such that Bcl-2–Bax dissociate and apoptosis is induced.

## SUMMARY AND CONCLUSIONS

In recent years, researchers have made great advances elucidating the JNK signal pathway. A large number of JNK substrates and regulatory molecules have been discovered, creating a solid foundation for further research regarding the physiological and pathological functions of the JNK signal pathway. However, the JNK signal pathway is extremely complicated; to date little is known about its function in autophagy. Some important objectives in autophagic regulatory mechanism research should be: (1) Investigation of the relationship between JNK and various ATG genes and its impact on autophagy, by means of genetic methods and protein interaction techniques; (2) Investigation of the cross-action between JNK pathway and other autophagy signalling pathways. The known JNK pathway molecular mechanisms may be helpful or a good starting point for this work. Autophagy is an evolutionarily conserved metabolic process in eukaryotes from yeast to mammals, most of mammals have homologues to the ATG genes. Further researches on JNK signalling pathway might provide new therapies for clinical disease treatment and is highly useful in discovery of new drug targets and screening of new drugs.

## Abbreviations

Ambra1: activating molecule in Beclin1-regulated autophagy
ATG: autophagy-related
Bcl-2: B-cell lymphoma 2
BH3 domain: Bcl-2-homology-3 domain
Bnip3: Bcl2/adenovirus E1B 19 kDa-interacting protein 3
Bif-1: Bax -interacting factor-1
BMK1: big MAP kinase 1
ca-FoxO3: constitutively active FoxO3
caspase: cysteine aspartate-special proteases
CCD: coiled-coil domain
CMA: chaperone-mediated autophagy
CML: chronic myeloid leukemia
Cvt: cytoplasm-to-vacuole targeting
dFoxO: drosophila FoxO
ECD: evolutionarily conserved domain
ERK: extracellular regulated kinases
FOX: forkhead box
JNK: c-Jun N-terminal kinase
Lc3: light chain 3
MAP1LC3: microtubule-associated protein 1 light chain 3
MAPK: mitogen-activated protein kinase
MEFs: Mouse Embryonic Fibroblasts
mTOR: mammalian target of rapamycin
PAS: phagophore assembly site
PCD: programmed cell death
PI3K: phosphoinositide 3-kinase
PKB: protein kinase B
RIP: receptor-interacting protein
SAPKs: stress-activated protein kinases
TNF-α: tumour necrosis factor-α
UVRAG: UV irradiation resistance-associated gene protein
Vps: vacuolar protein sorting
